# Accelerated protein digestion by numbering-up nanochannels, followed by LC–MS analysis

**DOI:** 10.1007/s44211-025-00867-w

**Published:** 2026-01-30

**Authors:** Fang-Yu Huang, Po-Yen Chen, Po-Yin Chen, Chihchen Chen, Kyojiro Morikawa

**Affiliations:** 1 Department of Power Mechanical Engineering, National Tsing Hua University, Hsinchu, 300044 Taiwan; 2 Institute of NanoEngineering and MicroSystems, National Tsing Hua University, Hsinchu, 300044 Taiwan; 3 Collaborative Research Organization for Micro and Nano Multifunctional Devices, The University of Tokyo, Tokyo, 113-8656 Japan; 4 Kanagawa Institute of Industrial Science and Technology, Kanagawa, 243-0435 Japan

**Keywords:** Nanofluidics, Nanochannel, Protein analysis, Enzymatic reaction, Trypsin

## Abstract

Techniques for analyzing proteins, including single-cell proteomics, are increasingly important and employed in diverse fields. The time-consuming nature of protein digestion presents a bottleneck in proteomics studies, however. To accelerate digestion reactions, reactors using packed beads or monolithic columns with immobilized enzymes are often used, but controlling the reaction remains challenging due to non-uniform gap sizes. We previously developed a picoliter nanofluidic digestion method, but comprehensive analysis of protein digestion using this method was difficult due to limited detection methods in the nanochannels. In this study, we developed a thin-layer nanofluidic device with a nanochannel width of 1.2 mm and depth of 310 nm to increase the product volume to the microliter scale. The nanofluidic device was successfully fabricated via APTES modification, vacuum ultraviolet patterning, washing, bonding, and enzyme immobilization. With the volume-up and numbering-up of the nanochannels, nL/min nanofluidic flow was observed and well controlled over a 16 min reaction. Digested cytochrome c in the nanochannels was collected at microliter scale, which enabled the use of conventional analyses such as liquid chromatography–mass spectrometry. Comparison of the reaction rates for a nanochannel-digested sample and a bulk-digested sample revealed 12–178 times faster reaction in the nanochannels, even when the digestion was performed in nanochannels under acidic conditions. The reason for the accelerated reaction rate remains unclear, but the unique properties of nanochannels may hold the key to elucidating the reaction mechanism.

**Graphical abstract:**

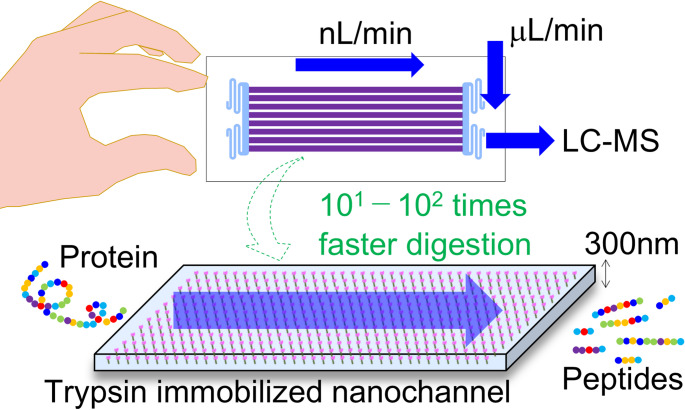

**Supplementary Information:**

The online version contains supplementary material available at 10.1007/s44211-025-00867-w.

## Introduction

The analysis of proteins, including single-cell proteomics studies [1–3], is increasingly important and employed in diverse fields, such as protein engineering [4, 5], cancer treatment [6, 7], and regenerative medicine [8, 9]. Understanding protein abundance and activity is critical for elucidating complex biological processes, including those involved in disease progression, cell differentiation, and fate determination [10]. Such knowledge is also essential for the development of therapies, vaccines, and diagnostics. Analyses of DNA and RNA can provide qualitative insights into gene expression, such as activation or repression under specific conditions, but such analyses cannot provide detailed information regarding protein abundance, cellular localization, post-translational modifications, or molecular interactions. This limitation highlights the need for techniques that enable direct protein analysis. A variety of analytical methods suitable for proteins have been developed, including gel electrophoresis [11], immunoassays [12], chromatography [13], and mass spectrometry (MS) [14]. Among these techniques, MS is particularly powerful because it enables the highly specific identification of proteins present in low concentrations. Recent notable advances in MS technology have greatly contributed to the realization of single-cell proteomics. In typical studies, comprehensive proteome analysis requires the digestion of proteins into peptides, followed by separation using techniques such as chromatography and electrophoresis.

Proteins of interest are usually present in complex mixtures of multiple types of molecules; however, as the quantities of different proteins in a sample can vary significantly, the two MS ionization techniques used for large molecules tend to either miss or suppress signals of more abundant proteins. In addition, proteins are often present in large quantities, which makes it difficult to visualize and identify them using MS. The digestion step is critically important in the process of MS analyses of proteins. During digestion, proteases are used to break down proteins into their constituent peptides. Traditional trypsin digestion typically requires 4 to 18 h for the enzyme to adequately cleave intact proteins into peptides. One approach involves the digestion of proteins using single slides placed in plastic Petri dishes under controlled conditions of high humidity and a pH of 7.4 maintained using ammonium bicarbonate at 37 °C[15]. In another method, protein-containing pieces of a dehydrated electrophoresis gel are incubated in alkaline bicarbonate solution to increase the pH, followed by overnight protease digestion at 37 °C. The resulting peptides are then extracted from the gel pieces into clean microcentrifuge tubes for further MS analysis [16].

Despite its widespread use, several inherent properties of trypsin pose challenges for MS analyses. Because it cleaves proteins at adjacent basic amino acids, trypsin also hydrolyzes its own peptide bonds upon activation, a process known as self-digestion. This self-digestion adversely affects the stability of the enzyme and also limits the potential digestion time and reduces digestion efficiency. Therefore, achieving optimal digestion in conventional trypsin-based methods remains a challenge. Immobilizing the enzyme at a high surface to volume (S/V) ratio has been shown to be effective for overcoming these limitations. Immobilization of the enzyme prevents self-digestion, and the use of a sufficiently high S/V ratio permits initiation of the enzymatic reaction between the target protein in the liquid phase and the surface-immobilized enzyme. A number of research groups have developed conventional reactors using packed beads [17, 18] or monolithic columns [19, 20] modified with enzymes on the surface of the packing material. However, it is difficult to control the reactions using these systems due to non-uniformity of the gaps between the beads or monolith material in the flow path. As a result, it is difficult to define the reaction time and concentration based on the unknown flow velocity and unknown enzyme density and surface area. In addition, low stability and low reproducibility [21] have been pointed out due to the particle leakage and fluctuations of pressure drop in the packed beads. To solve these issues, the reactors with well-controlled nanochannels, which are free from the issues of bead reactors, have been anticipated. To provide well-controlled nanochannels, our group developed nanofluidic enzymatic reactors in which the density of the enzyme on the nanochannel surface [22], as well as the enzyme’s stability [23] and kinetics [24], were well characterized, and we then used these reactors to demonstrate protein digestion [25]. This size-regulated nanochannel device enabled the measurement of both enzyme density and flow velocity; as a result, both the kinetics and stability of the enzyme could be determined. However, detailed protein analysis using this nanochannel system remained difficult due to the small size of the nanochannels (permitting only picoliter-scale sample volume). This limitation necessitated a detector in the nanochannel for in situ analyses, and due to the limitations of the detection tool, comprehensive analysis of protein digestion was difficult.

**Fig. 1:**
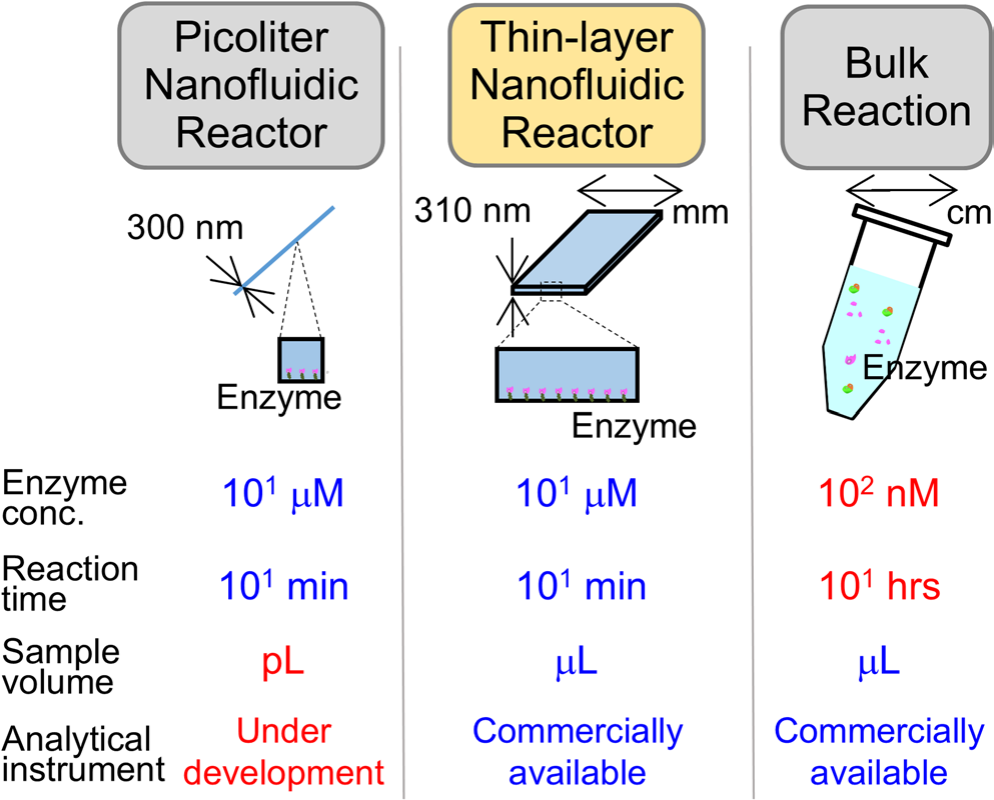
Concept of the thin-layer nanofluidic reactor, which enables ultra-fast reactions using microliter-scale samples for subsequent LC–MS analysis

In the present study, we developed a thin-layer nanofluidic device in which the nanochannel has a micrometer-millimeter width and nanometer-scale depth, enabling an increase in the product volume to the microliter scale, as shown in Fig. 1. In addition to volume-up of the nanochannel, the volume of sample that could be analyzed was increased by numbering-up of the nanochannels. This nanofluidic system enables the collection of samples of digested protein within the nanochannels on a microliter scale, which in turn enables the use of conventional analytical techniques such as liquid chromatography (LC)-MS. We then demonstrated ultra-fast protein digestion in the nanochannels by analyzing a digested sample using LC–MS.

## Design

**Fig. 2:**
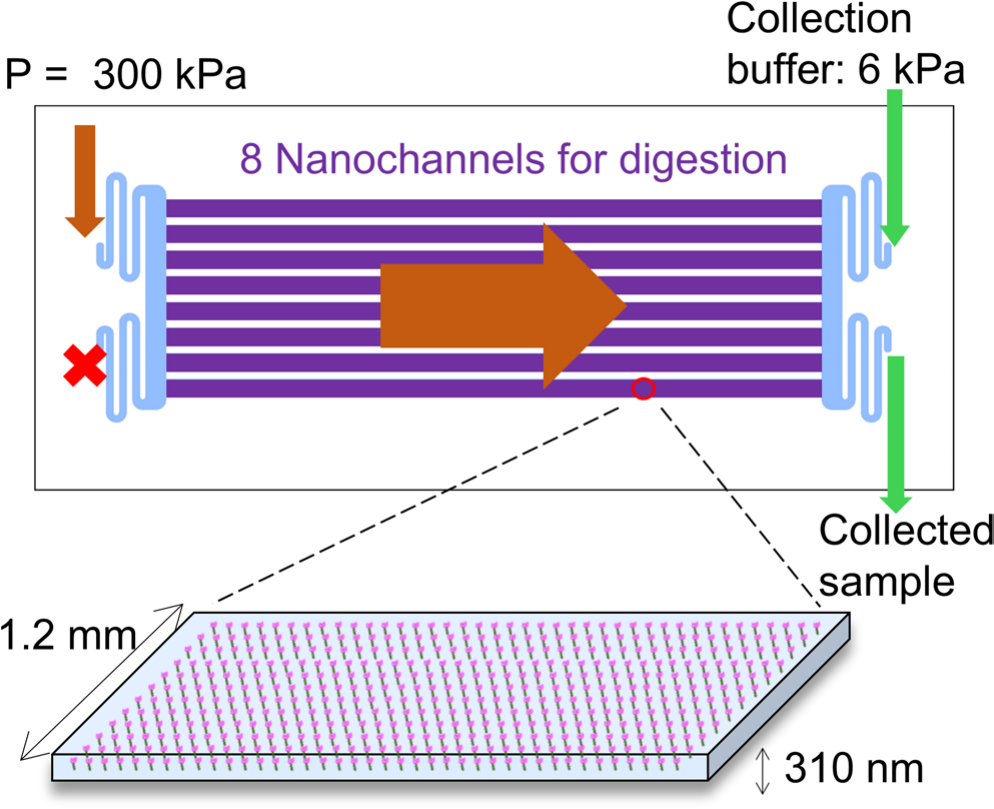
Design of the nanofluidic device, showing the direction of sample flow during digestion in the nanochannels and sample collection in the microchannels

The design of the nanofluidic device is illustrated in Fig. 2. The nanochannels (purple-colored channels in Fig. 2) used for protein digestion were designed to have a width of 1.2 mm, length of 50 mm, and depth of 310 nm. It should be noted that the several tens of times faster reaction in 300 nm channels was confirmed in our previous studies [22–25], and the acceleration was not confirmed in μm scale channels. Therefore, a depth of 310 nm was chosen in order to maintain the ultra-fast reaction rate demonstrated in our previous studies [22–25], and the width was expanded to 1.2 mm in order to permit an increase in the flow rate. Furthermore, in order to increase the scale of digested sample collection to the microliter level, the device was designed to have 8 nanochannels spaced 0.8-mm apart in a parallel configuration. The microchannels (blue-colored channels in Fig. 2), which had a depth of 20 mm, were placed at the inlet and outlet of the nanochannels, with two curved channels 0.5-mm wide and 24.36-mm long and a straight channel of 1.5-mm width and 16-mm length. In this design, the protein sample is introduced from the microchannel inlet (upper left) with closing of the microchannel outlet (lower left) by application of 300 kPa of pressure. Under this condition, the flow rate within the 8 nanochannels was calculated at 8.5 nL/min, and the passage time (i.e., reaction time) in the nanochannels was therefore 18 min at a flow velocity of 48 μm/s. In our previous results [25] suggested digested peptide peaks in 15 min digestion, therefore, a similar digestion time was designed in this study. The bottom surface of the nanochannels contained immobilized trypsin, and digestion occurred through interaction between the protein molecules in the liquid phase and the trypsin molecules immobilized on the nanochannel surface. A microchannel for sample collection was connected at the outlet of the nanochannels. The collection buffer was introduced from the microchannel inlet (upper right) by application of 6 kPa of pressure, and the sample was then collected from the microchannel outlet (lower right) at a rate of 2.2 μL/min.

## Experimental

### Nanofluidic device fabrication

The procedures for fabrication of the nanochannels and microchannels on a fused silica substrate (70 × 30 mm, 0.7-mm thick, VIOSIL-SQ, Shin-Etsu Chemical Co., Ltd., Japan) were described in our previous reports [26–29]. Briefly, the nanochannels and microchannels were patterned using lithography and formed by dry etching. After the nanochannel fabrication, the channel was characterized by a surface profiler (Dek-tak XT Stylus Profilometer, Bruker Taiwan Co., Ltd, Zhubei, Taiwan). The entire substrate with nanochannels was modified using (3-aminopropyl)triethoxysilane (APTES; 99%, cat. #: 919-30-2, Thermo Scientific, USA), and the APTES was patterned using vacuum ultraviolet (VUV) irradiation [22–27]. After VUV irradiation, the glass substrates with microchannels and APTES-patterned nanochannels were bonded together. In contrast to the previous APTES and VUV patterning method, the VUV light irradiation time was increased to 30 min. In addition, to ensure a cleaner bond, the solvent used for the washing step after VUV irradiation was changed to the following order: isopropanol (IPA), ethanol, and pure water. After bonding, the device was baked at 110 °C for 3 h.

### Enzyme immobilization

Enzyme immobilization was also carried out according to our previous reports, based on the trypsinogen immobilization method [22–25], especially to keep the same enzyme density and apparent enzyme concentration as in previous work [22]. Briefly, poly(ethyleneglycol) ([PEG] silane PEG, MPEG-SILANE, 5000 DA, cat. #: 25322-68-3, Echo Chemical Co., Ltd.) was prepared at a DI water to ethanol (≥ 99.8%, cat. #: 64-17-5, Honeywell, Germany) ratio of 0.1 wt%. It was introduced into the nanochannels via the microchannels to prevent protein adsorption in the SiOH group region. For crosslinking, 2.5% glutaraldehyde solution (~ 50% water, cat. #: 111-30-8, AccuStandard) was prepared in 100 mM borate buffer (pH 8.0). The borate buffer was formulated using boric acid (cat. #: 10043-35-3, Sigma-Aldrich) and 1 M sodium hydroxide (NaOH, cat. #: 1310-73-2, Honeywell, Germany). This solution was introduced into the nanochannels to start crosslinking with the amino group on the APTES surface. A solution of trypsinogen from bovine pancreas (2 mg/mL; cat. #: T1143-250MG, Sigma-Aldrich) in 100 mM borate buffer containing 20 mM calcium chloride (CaCl_2_; cat. #: 10043-52-4, Sigma-Aldrich) was prepared and introduced into the nanochannels to prevent self-digestion during the immobilization process. This process was carried out at 4 °C to minimize unwanted enzymatic activity and ensure optimal conditions for immobilization. A 0.01-unit solution of enterokinase was prepared in 100 mM borate buffer (pH 8.0) containing 20 mM Trizma hydrochloride (Tris–HCl; cat. #: 1185-53-1, Sigma-Aldrich), 2 mM calcium chloride (CaCl_2_; cat. #: 10043-52-4, Sigma-Aldrich), and 50 mM sodium chloride (NaCl; cat. #: 7647-14-5, Sigma-Aldrich). The enterokinase solution was introduced into the nanochannels to activate the trypsinogen and form trypsin.

### Nanofluidic protein digestion

Flow velocity was measured before conducting nanofluidic protein digestion experiments. A solution of 1 M sunset yellow FCF dye, content 90% (cat. #: 465224-25G, Sigma-Aldrich), was introduced into the nanofluidic device, and the flow velocity of each nanochannel was observed under a microscope.

For nanofluidic digestion experiments, cytochrome c from horse heart (2 mg/mL, cat. #: 9007-43-6, Sigma-Aldrich) was prepared in 95% DI water containing 5% acetonitrile (ACN, cat. #: 75-05-8, Honeywell, Germany) and 0.05% trifluoroacetic acid (TFA, 99%, cat. #: 76-05-1, Alfa Aesar). In addition, bradykinin (2.0 mg/mL, cat. #: 5979-11-3, Enzo) was introduced along with cytochrome c as an internal standard. The inclusion of bradykinin in the sample facilitated the normalization of peptide peak intensities, enabling more accurate and reliable quantification of the target protein [30]. Notably, the bioactive properties of bradykinin, primarily related to physiologic and pathologic processes such as vasodilation and inflammatory responses [31], have minimal impact on the enzymatic digestion of cytochrome c. The solution containing both cytochrome c and bradykinin was introduced into the nanochannels at 300 kPa, and the sample digested in the nanochannels was collected at the outlet microchannel at 6 kPa. The sample was collected at the microchannel using collection buffer (water containing 0.05% TFA).

For reference, bulk-scale digestion experiments were also performed. Trypsin solution (2 mg/mL, cat. #: T1426-250MG, Sigma Aldrich, USA) was prepared in a microcentrifuge tube containing 1 mL of borate buffer (20 mM Tris–HCl, 2 mM CaCl_2_, and 50 mM NaCl). Subsequently, 2 mg each of cytochrome c and bradykinin were added to the trypsin solution. The mixture was then incubated at room temperature for 12 h. The digested sample was then diluted 1,000-fold with borate buffer to adjust the concentration to a similar range as that of the sample digested in the nanochannels (theoretically, the sample digested in the nanochannels was diluted by the collection buffer 260-fold in the microchannel).

Both the nanochannel- and bulk-digested samples were analyzed using HPLC (Vanquish Flex UHPLC, Thermo Scientific) and high-resolution hybrid quadrupole-orbitrap mass spectrometry on a Q Exactive Plus hybrid quadrupole-orbitrap instrument (Thermo Scientific). Peptides were separated using an Accucore C8 column (SN #10586785). The mobile phase for peptide separation consisted of solvent A (water containing 0.1% formic acid; cat. #1.59013.4000, Supelco, Germany) and solvent B (a mixture of 80% ACN, 20% water, with 0.1% formic acid). Gradient elution was employed to achieve efficient resolution of peptides, starting from 95% solvent A and 5% solvent B (0–5 min) with a gradual change to 90% solvent A and 10% solvent B (5–6 min), followed by maintenance at 90% solvent A and 10% solvent B (6–15 min) and then a gradual change to 75% solvent A and 25% solvent B (15–16 min), maintenance at 75% solvent A and 25% solvent B (16–20 min), gradual change to 65% solvent A and 35% solvent B (20–30.3 min), gradual change to 35% solvent A and 65% solvent B (30.3–31 min), gradual change to 15% solvent A and 85% solvent B (31–40 min), gradual change to 5% solvent A and 95% solvent B (40–45 min), gradual change to 95% solvent A and 5% solvent B (45–46 min), and finally, maintenance at 95% solvent A and 5% solvent B (46–60 min).

## Results and discussion

**Fig. 3:**
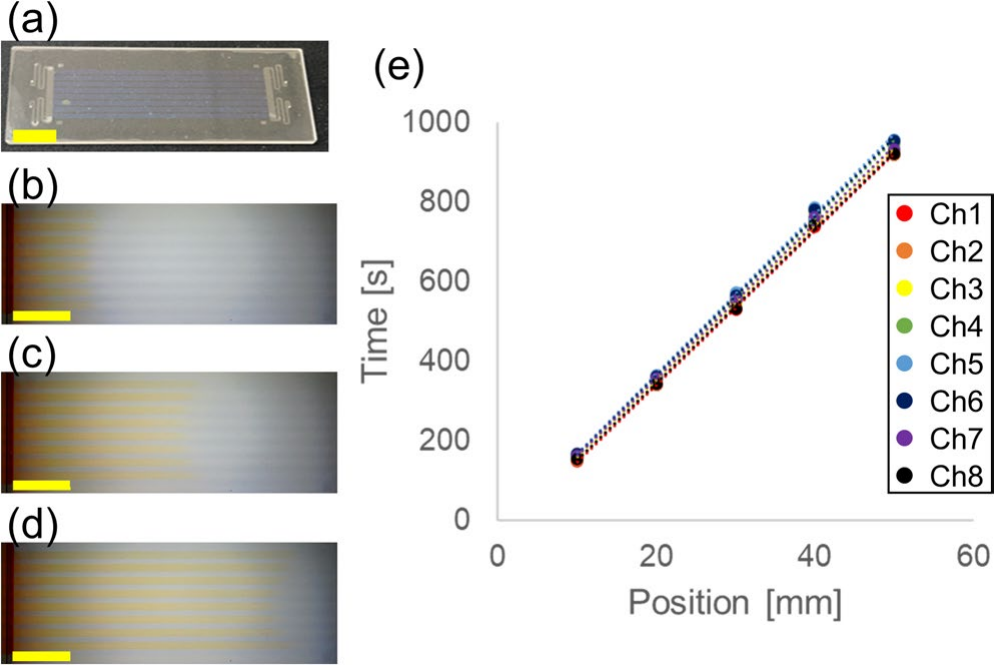
**a** Photograph of the fabricated nanofluidic device. Yellow bar = 10 mm. **b–d** Microscopic images at t = 200 s, 500 s, and 800 s. **e** Relationship between position and time for all 8 nanochannels

Figure 3a shows the fabricated nanofluidic device, and the characterized nanochannel result is shown in Figure S1. Due to the improved washing protocol, the device exhibited clean bonding. The silane-coupled amino group decomposed during VUV irradiation, and cleaning with IPA, ethanol, and pure water provided sufficient removal of degraded chemicals. The bonding energy, measured using the crack-opening method [26, 29, 32–36], was approximately 0.4 J/m^2^, sufficient for nanofluidic experiments on the order of 100 kPa [32]. The results of flow velocity measurements in the nanochannels are shown in Fig. 3b–d. All 8 nanochannels exhibited uniform transport, with an average velocity of 51 ± 1 µm/s, as determined from the slope of the lines shown in Fig. 3e and Figure S2. Based on this result, the liquid passage time in the nanochannels was estimated at 16 min, which corresponded to the reaction time for digestion.

**Fig. 4:**
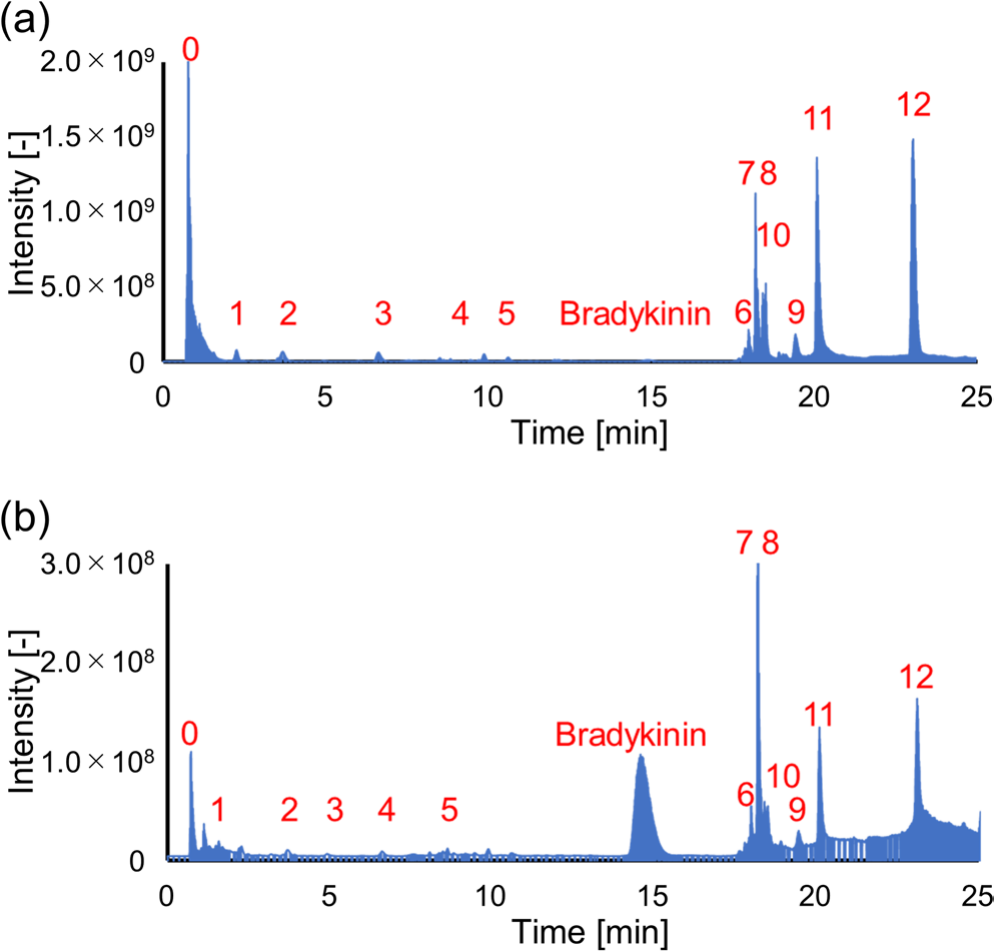
LC–MS chromatograms of digested samples. **a** Sample digested in bulk. **b** Sample digested in the nanofluidic device

Figure 4 shows the chromatograms of LC–MS analyses of the bulk-digested sample and sample digested in the nanofluidic device. Generally, trypsin cleaves peptide chains on the C-terminal site of lysine (K) and arginine (R) residues [37]. Peaks 0–12 were identified based on the results of an analysis of a standard sample of cytochrome c by Thermo Fisher [38, 39]. It should be noted that the bulk-digested sample included peptides derived from trypsin self-digestion, but generally, the trypsin peptide peaks did not overlap with cytochrome c peaks 0–12 considering the cleavage sites and sequences of both cytochrome c [38] and trypsin [40]. The relatively higher signals were observed in the bulk digestion because the concentrations of the produced peptides in the bulk digestion were higher due to the 12 h reaction, and the lower concentrations of them in the nanochannel digestion due to the much shorter 16 min reaction. In addition, in the chromatogram of the nanochannel digestion, an increased background was observed at the later retention time around 20–25 min. The detailed MS signals were shown in Figure S3, and additional signals in the high m/z region were observed in the digested sample in the nanochannels. One of the possible reasons is that the PEG molecules used in surface modification remained in the nanochannels, and the remaining PEG molecules were detected in the later retention time due to their hydrophobic interaction of methylene groups in PEG [41] in the reverse phase chromatography.

**Fig. 5:**
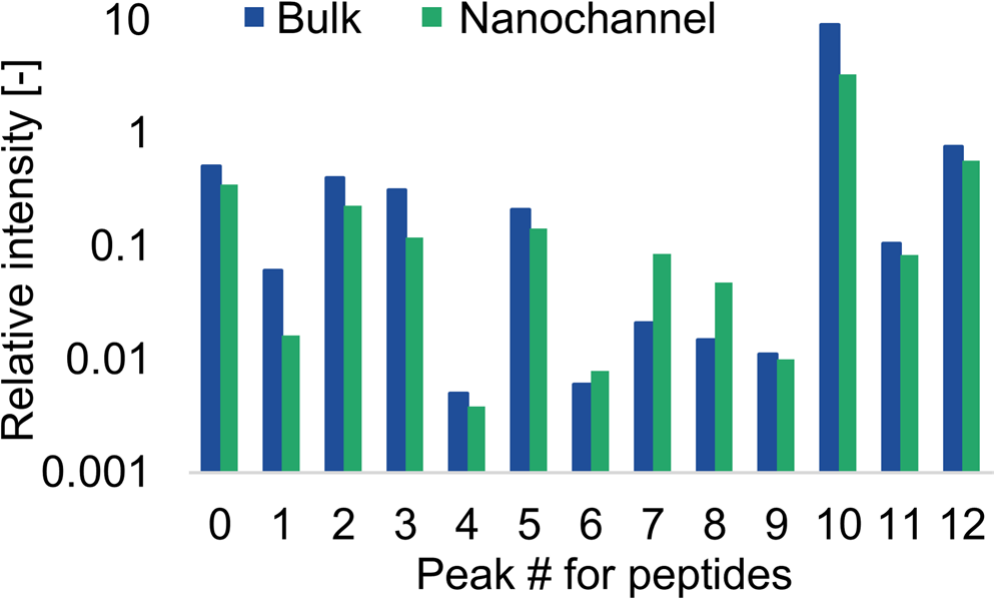
Signals for each peptide peak normalized against the internal standard signal

For comparison of the signal intensities, compensating for the different dilution rate between bulk and nanochannels, the signal intensities were normalized to those of bradykinin signals, as shown in Fig. 5. Notably, the intensity of almost all peaks was in a similar range for the bulk- and nanochannel-digested samples, and the signals of peaks 6, 7, and 8 were higher for the nanochannel-digested sample than the bulk-digested sample. Considering the reaction times of 12 h for the bulk digestion and 16 min for the nanochannel digestion, this result indicated that digestion in the nanochannels only for 16 min reached a similar or higher concentration of peptides obtained with bulk digestion for 12 h.

For fair comparison of produced peptides, each reaction rate of bulk and nanochannel was obtained by signal intensities per min. The reaction rates of the bulk and nanochannel digestion and the ratio of the reaction rate for each peak were summarized in Table 1. For all peaks, the reaction rate in the nanochannels was at least 10 times faster, and some peaks exhibited an acceleration on the order of 100 times faster than the bulk digestion. The digestion in the nanochannels in this study was performed under acidic conditions with TFA (measured pH = 2.2) to ensure smooth progression from the digestion occurring in the nanochannels, sample collection at the microchannels, and LC–MS measurements. The reason for this is that if we use the borate buffer for the nanochannel digestion, the borate buffer will be mixed with the TFA buffer at the outlet of the nanochannels, which induces the risk of clogging of the nanochannels by the produced salts. To prevent the risk, we used the TFA buffer for the digestion in the nanochannels. The optimal pH for trypsin activity is approximately 8.0, and the activity decreases markedly under acidic conditions [42–44]. The bulk digestion was performed at the optimal pH in the presence of borate buffer. In addition, trypsin was added in an almost equimolar amount for the bulk digestion to maximize reaction performance without minimizing the effects of self-digestion, although the typical concentration ratio of trypsin to target protein is 1:20 to 1:100 [45, 46] to prevent overlapping signals from self-digested trypsin peptides. Based on these points, the reaction condition for the bulk digestion was optimal, but the digestion in the nanochannels was much more rapid, even under the sub-optimal acidic condition.

**Table 1:** Summary for reaction rates and their ratio (nanochannel/bulk) for each peptide peak

Peak#	0	1	2	3	4	5	6	7	8	9	10	11	12
Bulk signal intensity/min	7.18 × 10^−4^	8.55 × 10^−5^	5.67 × 10^−4^	4.42 × 10^−4^	7.05 × 10^−6^	2.95 × 10^−4^	8.44 × 10^−6^	2.97 × 10^−5^	2.09 × 10^−5^	1.57 × 10^−5^	1.28 × 10^−4^	1.48 × 10^−4^	1.07 × 10^−3^
Nanochannel signal intensity/min	2.18 × 10^−2^	1.01 × 10^−3^	1.40 × 10^−2^	7.38 × 10^−3^	2.39 × 10^−4^	8.88 × 10^−3^	4.88 × 10^−4^	5.29 × 10^−3^	2.93 × 10^−3^	6.24 × 10^−4^	2.07 × 10^−1^	5.19 × 10^−3^	3.53 × 10^−2^
Reaction rate ratio	30	12	25	17	34	30	58	178	140	40	16	35	33

Electrostatic interaction with a negatively charged aspartic acid (D) residue in the substrate-binding pocket of trypsin [47] plays a key role in the cleavage of positively charged R and K peptide bonds in target proteins [48]. Our results showed the accelerated digestion rate in the nanochannels for all cleavage sites, especially continuous sequences of R-K or K-K, which correspond to peaks 6, 7, 8, 9, 11, and 12. These accelerations could have been related to electrostatic interaction because the nanochannels represent the dominant source of surface charges including an electric double layer [49–51] due to their extremely high surface area to volume ratio. In other words, electrostatic interactions with the surface, including electric double layer, may be more pronounced in nanoscale applications. Therefore, the nanochannel surface charge should also be taken into account. Considering the isoelectric points of trypsin (approximately 10–11 [52, 53]) and aspartic acid (approximately 2.77 [54, 55]), both the trypsin molecule and its aspartic acid residues should be positively charged under the acidic condition (pH = 2.2) employed in this study. However, some previous studies have reported a unique property change in the nanochannels, that is, a shift in isoelectric point (toward maintaining a negative surface) [56, 57] and/or an acceleration of hydrolysis [58] due to a unique proton mobility. These unique properties of the nanochannels may be the key to explaining the accelerated reactions observed in this study. Overall, we observed accelerated protein digestion even under the acidic condition. The reason for this acceleration remains unclear, but elucidating the unique properties of the nanochannels could provide the key to clarifying the reaction mechanism. In the future, with different digestion times, different nanochannel depths to achieve different apparent enzyme concentrations, different protein concentrations, including the reproducibility checking, the kinetics of the protein digestion in the nanochannels can be discussed, and clarification of the reaction mechanism in the nanochannel can be expected. Especially for the different protein concentration, a more accelerated reaction rate in the nanochannels can be expected when a lower concentration of the protein is introduced, because a reaction rate will be basically higher when the substrate concentration is closer to the enzyme concentration. Considering the general substrate concentration is in the sub-mg/mL or µM order [59–61], the concentration of cytochrome c in this study was relatively high, and a faster reaction can be highly anticipated with decreasing the substrate concentration.

## Conclusion

We developed a thin-layer nanofluidic device consisting of nanochannels with a width of 1.2 mm and depth of 310 nm to increase the product volume to the microliter scale. The nanofluidic device was successfully fabricated using APTES modification, VUV patterning, washing, bonding, and enzyme immobilization. Due to the volume-up and numbering-up of the nanochannels, nL/min nanofluidic flow was observed and could be well controlled over the course of the 16-min reaction. Cytochrome c digested in the nanochannels was collected at microliter scale, which enabled the use of conventional LC–MS analysis of the digestion product. The peaks of digested peptides were confirmed and the signals were normalized against the signals of an internal standard. Comparison of the reaction rate of the nanochannel-digested sample to the bulk-digested sample revealed that the reaction rate in the nanochannels was 12–178 times faster than the bulk digestion, even though the nanochannel digestion was performed under acidic conditions. The reason for the acceleration in reaction rate remains unclear, but the unique properties of the nanochannels may hold the key to clarifying the reaction mechanism.

## Supplementary Information

Below is the link to the electronic supplementary material.

Supplementary material 1 (Pdf 462 KB)

## Data Availability

Data will be made available on reasonable request.
