# Supplementary material for: Accelerated protein digestion by numbering-up nanochannels, followed by LC–MS analysis
Source: Anal Sci. 2026 Jan 30;42(7):501–9. doi: 10.1007/s44211-025-00867-w (PMC13294331; doi:10.1007/s44211-025-00867-w)
Supplement: Supplementary file 1 — Supplementary material 1 (Pdf 462 KB) [file 44211_2025_867_MOESM1_ESM.pdf]

Supplementary Information

Accelerated protein digestion by numbering-up nanochannels, followed by LC-MS analysis

Fang-Yu Huang[1], Po-Yen Chen[2], Po-Yin Chen[1,2], Chihchen Chen[1,2], Kyojiro Morikawa[1,2,3,4,*]

[1] *Department of Power Mechanical Engineering, National Tsing Hua University, Hsinchu 300044, Taiwan*

[2] *Institute of NanoEngineering and MicroSystems, National Tsing Hua University, Hsinchu 300044, Taiwan*

[3] *Collaborative Research Organization for Micro and Nano Multifunctional Devices, The University of Tokyo, Tokyo 113-8656, Japan*

[4] *Kanagawa Institute of Industrial Science and Technology, Kanagawa 243-0435, Japan*

*To whom correspondence should be addressed:
Kyojiro Morikawa: morikawa@pme.nthu.edu.tw

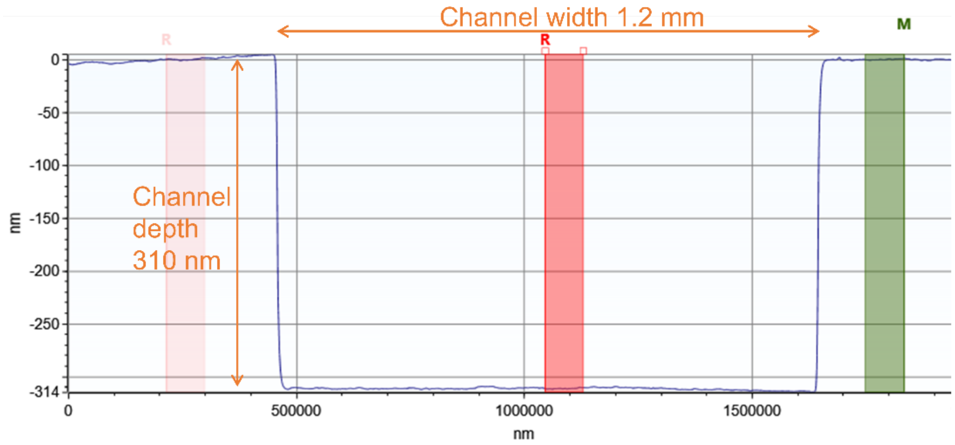

Figure S1. The fabricated nanochannel observed by a surface profiler. Blue line is the scanned results for the fabricated nanochannel.

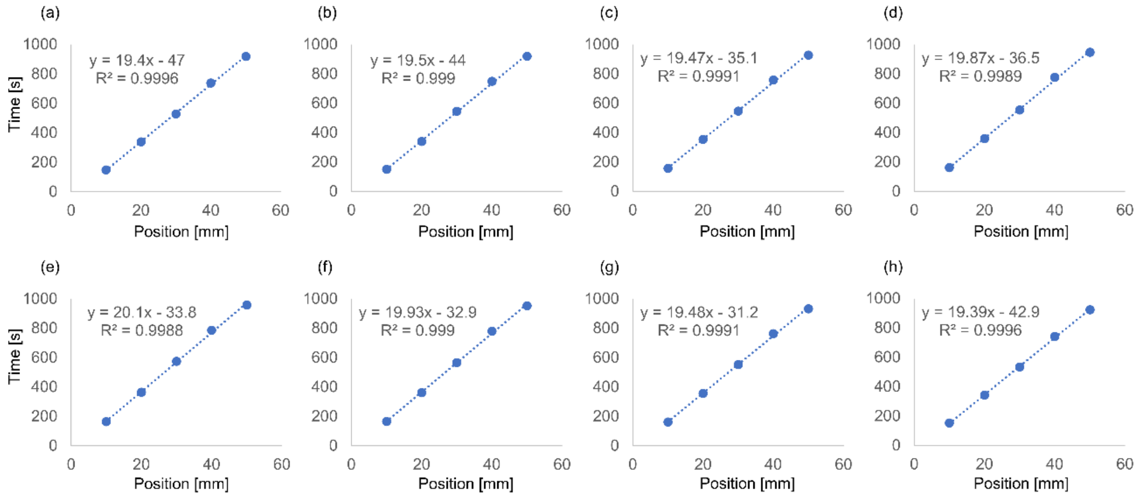

Figure S2. Relationship between position and time for all 8 nanochannels. (a) channel 1, (b) channel 2, (c) channel 3, (d) channel 4, (e) channel 5, (f) channel 6, (g) channel 7, (h) channel 8.

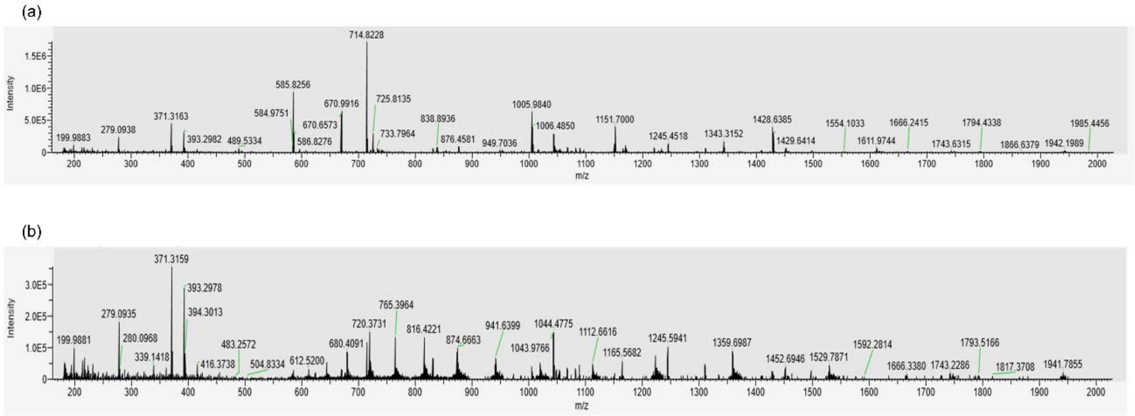

Figure S3. The MS signals at 23.9 min for retention time in the chromatogram in Figure 4. (a) bulk digestion, (b) nanochannel digestion.
